# Intracoronary acetylcholine testing among 746 consecutive Japanese patients with angina-like chest pain and unobstructed coronary artery disease

**DOI:** 10.1093/ehjopen/oeab012

**Published:** 2021-08-11

**Authors:** Shozo Sueda, Tomoki Sakaue

**Affiliations:** 1 Department of Cardiology, Ehime Prefectural Niihama Hospital, Hongou 3 choume 1-1, Ehime Prefecture, Niihama City 792-0042, Japan; 2 Department of Cardiology, Yawatahama City General Hospital, Ohira 1-638, Ehime Prefecture, Yawatahama City 796-8502, Japan

**Keywords:** Epicardial spasm, Coronary microvascular spasm, Acetylcholine testing, Japanese population

## Abstract

**Aims:**

Intracoronary acetylcholine (ACh) testing is useful for the detection of epicardial spasm (ES) and coronary microvascular spasm (CMS). We retrospectively analysed the incidence of ES and CMS in consecutive Japanese patients with unobstructed coronary artery disease.

**Methods and results:**

From January 1991 to February 2019, we performed intracoronary ACh testing of 1864 patients. Among these patients, a total of 746 consecutive patients (254 women, mean age 64 ± 11 years) who underwent first diagnostic angiography for suspected myocardial ischaemia and had unobstructed coronary arteries (<50%) were enrolled. Epicardial spasm was defined as ≥90% stenosis and usual chest symptoms and ischaemic ECG changes, while CMS was defined as <75% stenosis and usual chest symptoms and ischaemic ECG changes. We performed intracoronary ACh testing on both coronary arteries in 96% (716/746) of all subjects. Overall, ES was found in 329 patients (44%), whereas CMS was revealed in 40 patients (5%) including 4 patients with coexisting ES. In patients with ES, women made up 22%, and approximately three-quarters of the patients had resting chest pain. In contrast, women composed 65% (26/40) of those with CMS, and 15 patients with CMS had another chest symptom. Coronary microvascular spasm was frequently observed in the left coronary artery (LCA) but not the right coronary artery. Electrical cardioversion was necessary for two patients.

**Conclusions:**

Coronary microvascular spasm was recognized in only 5% of consecutive Japanese patients with unobstructed coronary artery disease, whereas ES was revealed in 44% of those patients. Coronary microvascular spasm was often observed in women and in the LCA.

## Graphical Abstract

**Graphical Abstract:**
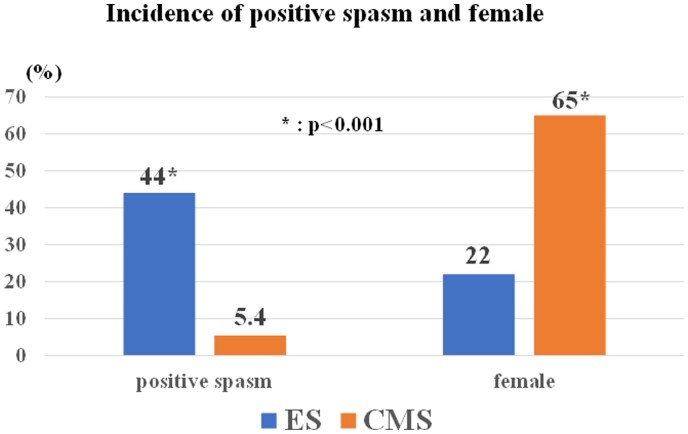

## Introduction

Coronary microvascular dysfunction is frequently observed in Caucasian populations with unobstructed coronary artery diseases.^1^^,^^2^ Furthermore, clinical outcomes in patients with coronary microvascular dysfunction and unobstructed coronary artery disease are not so favourable, although these patients have non-significant organic stenosis.^3^^,^^4^ According to previous reports, intracoronary acetylcholine (ACh) testing provoked epicardial spasm (ES) and coronary microvascular spasm (CMS) in a sizable number of patients with unobstructed coronary arteries.^5^^,^^6^ In contrast, ES is frequently recognized in Asian populations by performing spasm provocation tests using ACh and ergonovine.^7^^,^^8^ However, the incidence of CMS in eastern Asian and Japanese populations undergoing intracoronary ACh testing is uncertain. In this article, we retrospectively investigated the epicardial and microvascular spasm frequency in Japanese patients with new-onset cases who had unobstructed coronary arteries and were suspected of having myocardial ischaemia when we performed intracoronary ACh testing according to the Japanese Circulation Society (JCS) guidelines.^9^

## Methods

### Study patients

**Figure 1:**
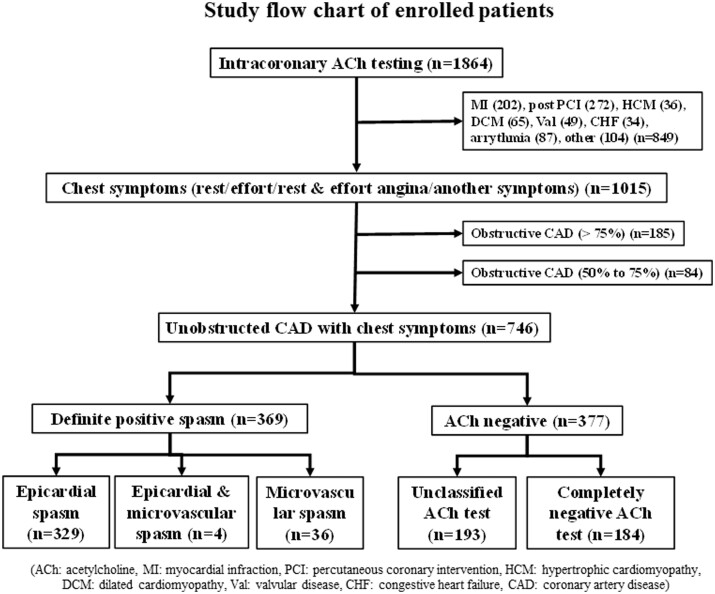
Study flow chart of enrolled patients.

From January 1991 to February 2019, we performed a total of 8351 coronary angiography procedures, including 2353 percutaneous coronary interventions and 5998 diagnostic and follow-up cardiac catheterizations. During the same time, we evaluated 986 patients undergoing coronary arteriography with a diagnosis of suspected acute myocardial infarction (AMI). Atherothrombotic AMI was recognized in 904 patients. Takotsubo syndrome was diagnosed in 16 patients and myocarditis was suspected in 5 patients. Myocardial infarction with non-obstructive coronary arteries was observed in 61 patients. We defined AMI as acute chest pain (chest pain at rest >30 min within the last 48 h) together with ECG changes suggesting myocardial ischaemia and/or elevation of cardiac markers. We also defined positive cardiac markers as being more than two-fold higher than the normal range of creatinine phosphokinase (>488 IU/L) or creatinine phosphokinase MB type (>32 IU/L). During the same period, we performed intracoronary ACh testing of 1864 patients. As shown in *Figure  1*, we enrolled 746 consecutive patients who underwent their first diagnostic angiography for suspected myocardial ischaemia without AMI and had unobstructed coronary arteries (<50%). According to the patient’s chest symptoms, we classified the study subjects into four groups: predominantly chest pain at rest, predominantly exertional chest pain, resting and exertional chest pain, and another chest symptom (not typical chest pain but suspected to be myocardial ischaemia). We excluded 849 patients, including 202 patients with myocardial infarction, 272 patients with post percutaneous coronary intervention, 36 patients with hypertrophic cardiomyopathy, 65 patients with dilated cardiomyopathy, 49 patients with valvular heart diseases, 34 patients with congestive heart failure, 87 patients with arrhythmias, and 104 patients with other conditions. We also excluded 269 patients with obstructed coronary arteries, including 185 patients with >75% stenosis and 84 patients with 50–75% stenosis. The provocation test was not performed if patients had heart failure (New York Heart Association functional class III or IV), renal failure (creatinine >2.0 mg/dL), or if isosorbide dinitrate was initially used to relieve a spasm in the coronary artery tested. The previous medical history was derived from patient medical records, including classical coronary risk factors. Hypertension was considered positive if the patient had already been treated with antihypertensive medicine or had a blood pressure of 140/90 mmHg, while diabetes mellitus was considered positive if the glycohaemoglobin was over 6.5% (National Glycohemoglobin Standardization Program) or was already being treated with a hypoglycaemic agent. Dyslipidaemia was considered positive if the patient had already been treated with statins or had a greater than 140 mg/dL low-density lipoprotein cholesterol. A history of smoking was considered positive if the patient had a history of smoking for at least 5 years in the past or was a habitual current smoker.

### Study protocol

The study protocol complied with the Declaration of Helsinki. Written informed consent was obtained from all patients before performing the ACh spasm provocation tests, and the protocol of this study was in agreement with the guidelines of the ethics committee at our institution. Cardiovascular medicines (calcium channel blockers, nitrates, nicorandils, and beta-blockers) were discontinued 24 h before coronary angiography, and sublingual nitroglycerine was also discontinued ≥4 h before the study. Cardiac catheterization was performed from 9:00 a.m. to 4:00 p.m. in the fasting state. Blood pressure and heart rate were continuously monitored, and a standard 12-lead electrocardiogram was recorded every 30 s during ACh testing. We read the ECG findings when ACh, saline and contrast medium were not injected into the responsible vessels for at least 60 s. We listened to the chest symptoms during ACh testing from all patients in detail and classified their chest symptoms into usual or unusual chest symptoms. A temporary pacemaker was inserted into the right ventricle of each ACh testing patient, and the pacing rate was set at 40–45 beats/min.

### Acetylcholine testing

Provocation of coronary artery spasm was performed with an intracoronary injection of ACh, as previously reported.^10^^,^^11^ Acetylcholine chloride was injected in incremental doses of 20, 50, and 80 µg into the right coronary artery (RCA) and of 20, 50, 100, and 200 µg into the left coronary artery (LCA) over 20 s with at least a 3-min interval between each injection. We have employed maximal 200 µg ACh since Aug 2012.^12^ Coronary arteriography was performed when ST-segment changes and/or, chest pain occurred or 1–2 min after the completion of each injection. When an induced coronary spasm did not resolve spontaneously within 3 min after the completion of ACh injections or when haemodynamic instability occurred as the result of coronary spasm, 2.5–5.0 mg of isosorbide dinitrate was injected into the involved vessel. After the spasm provocation tests were completed, an intracoronary injection of 5.0 mg isosorbide dinitrate was administered, and coronary arteriography was then performed in multiple projections.

### Assessment of acetylcholine testing

In the present study, the coronary arteriograms were analysed separately by two independent observers, but not blinded. The per cent luminal diameter narrowing of the coronary arteries was measured using an automatic edge-counter detection computer analysis system. The size of the coronary catheter was used to calibrate the images in millimetres, and the measurement was performed in the same projection of the coronary angiography at each stage. We defined positive ES as ≥90% transient stenosis and usual chest symptoms and ischaemic ECG changes.^13^ We also defined positive CMS as <75% transient stenosis and usual chest symptoms and ischaemic ECG changes.^14^ The degree of ST-segment depression was measured 80 ms after the J point. We considered a result to be positive when at least one of the following ischaemic ECG changes was demonstrated during and/or after the ACh test: (i) ST-segment elevation of ≥ 0.1 mV in at least two contiguous leads; and (ii) ST-segment depression of 0.1 mV in at least two contiguous leads. We also considered negative U waves as positive ischaemic ECG changes. Focal spasm was defined as a discrete transient vessel narrowing ≥90% localized in a major coronary artery, whereas diffuse spasm was diagnosed when transient vessel narrowing ≥90%, compared with baseline coronary angiography, was observed in ≥2 adjacent coronary segments of epicardial coronary arteries. We defined ACh low dose as ACh 20 µg and ACh high dose as ACh 80 µg in the RCA and ACh 200 µg in the LCA. The remaining doses of ACh were defined as ACh mid-dose. Spasm provoked sites were classified according to the American College of Cardiology (ACC)/American Heart Association (AHA) classification. An obstructed coronary artery was defined as ≥50% luminal narrowing according to the ACC/AHA classification.^15^ Proximal spasm was defined as coronary vasoconstriction observed in segments 1, 5, 6, or 11, while mid-vessel spasm was recorded when occurring in segments 2, 3, or 7. Distal spasm was also defined as vasoconstriction in segments 4, 8, 9, 10, and 12–15. We defined hypoplastic RCA as a very small RCA when the RCA supplies only to the right ventricle and ends before reaching the crux of the heart and prior to splitting off the posterior descending artery and posterolateral artery.^16^ We did not perform ACh testing in the hypoplastic RCA.

### Statistical analysis

Data analysis was carried out with SPSS (version 22.0, IBM Japan, Ltd, Tokyo, Japan). All data are presented as the mean ± 1 SD. Clinical characteristics, including coronary risk factors, and provoked spasm incidence, were analysed by Fisher’s exact test with correction or the Mann–Whitney *U* test. Multiple logistic regression analysis was performed by using forward variable selection based on the likelihood ratios to identify predictors of a positive ACh test and for the identification of patients with ES in comparison with those with CMS. The ratio of ES to CMS is presented as the median with an interquartile range (minimum to maximum). *P*-values <0.05 were considered to be significant.

## Results

### Overall results

As shown in *Table 1*, among 746 patients, the ACh test revealed definite positive spasms in 369 patients (49%), whereas the remaining 377 patients (51%) had negative ACh tests. Women comprised 34% of the study subjects, and the mean age was 64 years old. Resting chest pain was recognized in 57% of the study subjects, and another chest symptom was observed in 19%. We skipped intracoronary ACh testing in 29 RCAs, including 19 vessels due to hypoplastic artery, 6 vessels that were difficult to insert the RCA ostium into (catheter-induced spasm: 3, 4 French catheter wedge: 1, semiselective engagement: 2), and in 4 vessels after the administration of nitroglycerine to relieve an ES of the LCAs. Furthermore, we could not perform intracoronary ACh testing in one LCA after the relief of a prolonged RCA ES. We could perform intracoronary ACh testing on both coronary arteries in 96% (716/746) of the study subjects. We could also not analyse five ECG changes during ACh testing due to a pacing rhythm. Two patients with definite microvascular spasm in the LCA who complained of usual chest symptoms were classified as unclassified ACh tests because we could not detect ischaemic ECG changes due to pacing rhythm in the RCAs. Three patients (RCA: 2 and LCA: 1) with the occurrence of usual chest symptoms and ≥90% coronary constriction were classified as unclassified ACh tests because we could not detect ischaemic ECG changes due to pacing rhythm during the ACh tests.

**Table 1:** All patient clinical characteristics

	All patients	ACh definite positive	ACh negative	*P* **-value**
Number	746	369	377	
Sex (female)	254 (34)	99 (27)	155 (41)	<0.001
Age, year, mean ± SD	64 ± 11	65 ± 10	64 ± 11	0.9907
Follow-up duration month, mean ± SD	50 ± 32	48 ± 32	52 ± 32	0.5446
Type of chest symptom				
Resting chest pain	424 (57)	252 (68)	172 (46)	<0.001
Exertional chest pain	89 (12)	43 (12)	46 (12)	0.8172
Effort and resting chest pain	92 (12)	51 (14)	41 (11)	0.2211
Another chest symptom	141 (19)	23 (6)	118 (31)	<0.001
ACh spasm testing				
Left coronary artery	745 (99)	368 (99)	377 (100)	0.3117
Right coronary artery	717 (96)	347 (94)	370 (98)	0.0037
Both coronary	716 (96)	346 (94)	370 (98)	<0.01
LVEF by UCG (%), mean ± SD	67 ± 8	67 ± 9	68 ± 7	0.7885
Coronary risk factors				
Hypertension	293 (39)	141 (38)	152 (40)	0.5557
Dyslipidaemia	330 (44)	172 (47)	158 (42)	0.1960
Diabetes mellitus	149 (20)	70 (19)	79 (21)	0.4978
History of smoking	472 (63)	266 (72)	206 (55)	<0.001
Medications before ACh testing				
Calcium channel blocker	390 (52)	237 (64)	153 (41)	<0.001
Nitrate or nicorandil	293 (39)	185 (50)	108 (29)	<0.001
Beta-blocker	52 (7)	21 (6)	31 (8)	0.1745
ACEI or ARB	102 (14)	59 (16)	43 (11)	0.0684
Statin	138 (18)	79 (21)	59 (16)	0.0428

ACEI, angiotensin-converting enzyme inhibitor; ACh, acetylcholine; ARB, angiotensin receptor blocker; LVEF, left ventricular ejection fraction; UCG, ultrasound cardiography.

### Epicardial spasm

A summary of the results from each patient is shown in *Table 2*. Just ES was provoked in 329 patients. Women made up 22% of these patients, and approximately three-quarters of patients had resting chest pain. As shown in *Table 3*, a diffuse spasm was found in 66%, whereas a segmental spasm was revealed in 34%. There were no differences between the provoked spasm sites and the morphological configuration except for mid-vessel spasm in the RCA. The incidence of focal or subtotal/occluded spasms in the mid-vessel RCA was remarkably higher than that of diffuse spasms (64% vs. 36%, *P* < 0.001). Distal spasm was provoked at a high ACh dose compared with a low dose of ACh, as shown in *Table 3*. Definite positive ES in the RCA was observed in 247 patients, including 133 with ST-segment elevation and 114 with ST-segment depression, while 221 patients with ES in the LCA showed ischaemic ECG changes, including 90 with ST-segment elevation and 131 with ST-segment depression. Usual and unusual chest symptoms in the RCA were reported by 264 and 6 patients, respectively, whereas, in the LCA, unusual chest symptoms were observed in 13 patients, and usual chest symptoms were found in 257 patients. One-vessel ES was revealed in 165 patients (50%), whereas multiple ES was diagnosed in 164 patients (50%), including 88 patients (27%) with two-vessel spasms and 76 patients (23%) with triple vessel spasms.

**Table 2:** All patient clinical characteristics

	Epicardial spasm	Epicardial and microvascular spasm	Microvascular spasm	Unclassified ACh test	ACh complete negative
Number	329	4	36	193	184
Sex (female)	73 (22)^***^	1 (25)	25 (69)	60 (31)^***^	95 (52)^*^
Age, year, mean ± SD	64 ± 10	63 ± 7	69 ± 12	64 ± 12	64 ± 10
Follow-up duration, month, mean ± SD	48 ± 31	30 ± 22	50 ± 35	50 ± 33	53 ± 32
Type of chest symptom					
Resting chest pain	242 (74)^***^	3 (75)	7 (19)	112 (58)^***^	60 (33)
Exertional chest pain	33 (10)^**^	0	10 (28)	24 (12)^*^	22 (12)^*^
Effort and resting chest pain	46 (14)	0	5 (14)	29 (15)	12 (7)
Another chest symptom	8 (2)^***^	1 (25)	14 (39)	28 (15)^***^	90 (49)
ACh spasm testing					
LCA	328 (99)	4 (100)	36 (100)	193 (100)	184 (100)
RCA	311 (95)	4 (100)	32 (89)	186 (96)	184 (100)
Both coronary artery	310 (94)	4 (100)	32 (89)	186 (96)	184 (100)
One-vessel epicardial spasm	165 (50)	4 (100)	0	0	0
Two-vessel epicardial spasm	88 (27)	0	0	0	0
Three-vessel epicardial spasm	76 (23)	0	0	0	0
RCA hypoplastic artery	11 (3)	0	2 (6)	6 (3)	0
Difficult to insert into the RCA	4 (1)	0	1 (3)	1 (1)	0
Not enforced another vessel after NG relief	4 (RCA: 4)	0	1 (LCA: 1)	0	0
LVEF by UCG (%) mean ± SD	67 ± 9	68 ± 6	68 ± 8	68 ± 7	67 ± 7
Coronary risk factors					
Hypertension	124 (38)	1 (25)	16 (44)	74 (38)	78 (42)
Dyslipidaemia	159 (48)	1 (25)	12 (33)	90 (47)	68 (37)
Diabetes mellitus	60 (18)	1 (25)	9 (25)	42 (22)	37 (20)
History of smoking	251 (76)^***^	3 (75)	12 (33)	128 (66)^***^	78 (42)
Medications before ACh testing					
Calcium channel blocker	217 (66)	1 (25)	19 (53)	101 (52)	52 (28)^**^
Nitrate or nicorandil	174 (53)^**^	1 (25)	10 (28)	68 (35)	40 (22)
Beta-blocker	16 (5)	0	5 (14)	13 (7)	18 (10)
ACEI or ARB	47 (14)^*^	1 (25)	11 (31)	16 (8)^***^	27 (15)^*^
Statin	71 (22)	0	8 (22)	34 (18)	25 (14)

ACEI, angiotensin-converting enzyme inhibitor; ACh, acetylcholine; ARB, angiotensin receptor blocker; LCA, left coronary artery; LVEF, left ventricular ejection fraction; NG, nitroglycerine; RCA, right coronary artery; UCG, ultrasound cardiography.

* *P* < 0.05,

** *P* < 0.01,

*** *P* < 0.001 vs. microvascular spasm.

**Table 3:** Acetylcholine dose and epicardial spasm site and morphological configuration

	Number	Diffuse spasm	Focal spasm	Subtotal or occluded spasm	ACh low dose	ACh mid-dose	ACh high dose
LAD							
Proximal	81	61 (75)	9 (11)	11 (14)	16 (20)	59 (73)	6 (7)
Mid	84	57 (68)	9 (11)	18 (21)	9 (11)	68 (81)	7 (8)
Distal	41	32 (78)	0	9 (22)	1 (2)	29 (71)	11 (27)^#^
RCA							
Proximal	61	44 (72)	8 (13)	9 (15)	20 (33)	25 (41)	16 (26)
Mid	96	35 (36)^*^	16 (17)	45 (47)	34 (35)	37 (39)	25 (26)
Distal	90	60 (67)	6 (7)	24 (27)	13 (14)^#^	43 (48)	34 (38)^&^
LCX							
Proximal	79	68 (86)	6 (8)	5 (6)	12 (15)	61 (77)	6 (8)
Distal	38	20 (53)^*^	3 (8)	15 (39)	2 (5)	24 (63)	12 (32)^#^
All proximal	221	173 (78)	23 (10)	25 (11)	48 (22)	145 (66)	28 (13)
All mid	180	92 (51)^*^	25 (14)	63 (35)	43 (24)	105 (58)	32 (18)
All distal	169	112 (66)	9 (5)	48 (28)	16 (9)^*^	96 (57)	57 (34)^*^
All	570	377 (66)	57 (10)	136 (24)	107 (19)	346 (61)	117 (21)

LAD, left anterior descending artery; LCX, left circumflex artery; RCA, right coronary artery; LCA, left coronary artery; ACh low dose: 20 µg; ACh mid-dose: RCA 50 µg and LCA 50/100 µg; ACh high dose: RCA 80 µg and LCA 200 µg.

* *P* < 0.001 vs. other,

# *P* < 0.01 vs. other,

& *P* < 0.05 vs. other.

### Coronary microvascular spasm

As shown in *Table 2* and Supplementary material online, *Table S1*, CMS was observed in 40 patients, including 4 patients with coexisting ES. Among these, 65% were women (26/40), and 15 patients (38%) had another chest symptom. We performed intracoronary ACh testing on both coronary arteries in 37 (93%) of 40 patients. The incidence of CMS observed in the LCA was significantly higher than that in the RCA [75% (30 patients) vs. 35% (14 patients), *P* < 0.001]. Furthermore, 10 patients (25%) had microvascular spasms in just RCAs without LCAs. ST-segment elevation was observed in 4 patients, whereas the remaining 36 patients had ST-segment depression.

### Unclassified acetylcholine tests

We observed 193 patients (26%) with unclassified ACh tests among 746 patients. Moderate vasoconstriction (75% ≤ < 90% ) by ACh testing was observed in 45 patients, including 19 patients with resting chest pain, 8 with exertional chest pain, 10 with rest and exertional chest pain, and 8 with other chest symptoms. The remaining 148 patients had unclassified ACh tests, including 63 patients with ≥90% stenosis and usual chest symptoms without ischaemic ECG changes, 18 patients with ≥90% alone without usual chest symptoms or ischaemic ECG changes, 23 patients with usual chest symptoms alone, 13 patients with ≥90% stenosis and ischaemic ECG changes without usual chest symptoms, 16 patients with ≥90% stenosis and ischaemic ECG changes with unusual chest symptoms, 7 patients with unusual chest symptoms alone without ≥90% stenosis or ischaemic ECG changes, 3 patients with ≥90% and unusual chest symptoms without ischaemic ECG changes, 4 patients with ischaemic ECG changes without ≥90% or usual chest symptoms, and one patient with unusual chest symptom and ischaemic ECG changes without ≥90% narrowing. Furthermore, we found 184 patients (25%) with completely negative ACh results.

### Univariable analysis and multivariable analysis

As shown in Supplementary material online, *Table S2*, a history of smoking, the administration of calcium channel blockers and nitrate or nicorandil were the determinant factors between patients with epicardial and microvascular spasms and those with negative results. Multivariable analysis revealed significant differences regarding a history of smoking and the administration of nitrate or nicorandil between patients with ES and microvascular spasm as shown in Supplementary material online, *Table S3*.

### Complications

We experienced 20 complications (3%), including 11 non-sustained ventricular tachycardia, one sustained ventricular tachycardia, 5 blood pressure drops (<60 mmHg), 1 ventricular fibrillation, and 2 left main trunk spasm equivalents. Electrical cardioversion was necessary for two patients (one sustained ventricular tachycardia and one ventricular fibrillation). However, we had no irreversible complications such as myocardial infarction or a requirement for cardiac resuscitation. All 746 patients who had intracoronary ACh testing were discharged the next day without any complications.

## Discussion

In this study, we found a high frequency of ES and a low incidence of CMS in consecutive Japanese patients with unobstructed coronary arteries using intracoronary ACh testing based on JCS guidelines. We often observed diffuse spasms in each coronary artery except for mid-vessel spasms in the RCA. Focal or subtotal/occluded spasms were frequently observed in the mid-vessel of the RCA rather than diffuse spasms. The incidence of CMS in the LCA was markedly higher than that in the RCA, while a quarter of patients with CMS had microvascular spasms in just the RCA. There were only four CMS patients with coexisting ES. We could perform ACh tests without any irreversible complications, although electrical cardioversion was necessary for two patients.

### Clinical and angiographic characteristics of epicardial spasm and coronary microvascular spasm

**Figure 2:**
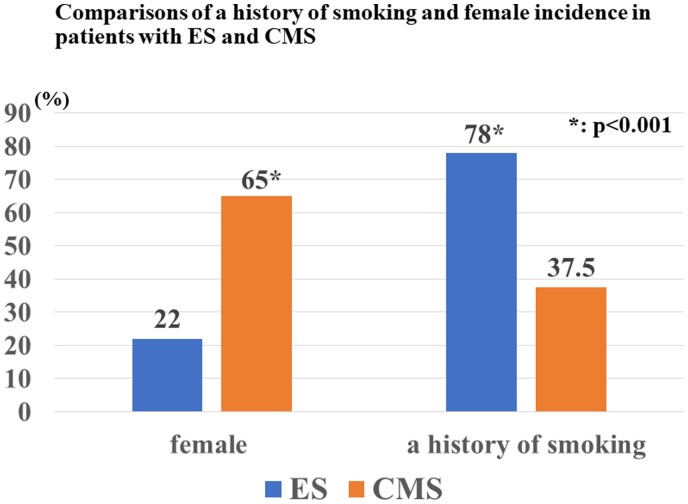
Comparisons of a history of smoking and female incidence in patients with epicardial spasm and coronary microvascular spasm.

Recently, similarities of coronary response by ACh testing between German and Japanese patients were reported.^17^ According to a European study, the most frequent type and location of inducible spasm by intracoronary ACh testing are distal and diffuse spasms.^18^ Furthermore, one-vessel spasm was observed in 67.4%, while multiple spasms were revealed in 32.6% (two-vessel spasms: 31.2% and three-vessel spasms: 1.4%). In this study, we recognized that the frequent spasm configuration was diffuse but not distal. There were no differences between provoked spasm sites and the morphological configuration except for mid-vessel spasm in the RCA. Especially in the mid-RCA, the incidence of focal or subtotal/occluded spasms was remarkably higher than that of diffuse spasms. This is one of the striking differences between European and Japanese populations. Furthermore, the incidence of single-vessel spasm in the European study was significantly higher than that in our study [67% (190/282) vs. 50% (165/329), *P* < 0.001], whereas the frequency of triple vessel spasms in this study was markedly higher than that in the European study [23% (76/329) vs. 1.4% (4/282), *P* < 0.001]. European CMS patients more often presented with exertional chest pain, while patients with CMS in this study presented with another chest symptom as well as exertional chest pain. As shown in *Figure  2*, the incidence of female in patients with CMS was markedly higher than that in those with ES (65% vs. 22%, *P* < 0.001), whereas a history of smoking was significantly higher in patients with ES than in those with CMS (78% vs. 37.5%, *P* < 0.001).

### Incidence of epicardial spasm and coronary microvascular spasm in Japanese populations compared with Western subjects

We previously reported the frequency of ES in Japanese consecutive patients with intracoronary injection of ACh,^19^ but we did not investigate the incidence of CMS in that cohort. In this study, we reanalysed the frequency of ES and CMS in patients with unobstructed coronary artery disease. We defined CMS as <75% stenosis by intracoronary ACh testing and usual chest symptoms and ischaemic ECG changes according to the Coronary Vasomotion Disorders International Study group definition.^14^ We diagnosed 40 patients (5.4%) with CMS, including four patients with coexisting ES. As shown in Supplementary material online, *Table S4*, according to the report by Mohri *et al.*,^20^ among 117 patients with chest pain and no flow-limiting (>50%) organic stenosis, ES was revealed in 63 patients (54%), and CMS was found in 29 patients (25%) by using intracoronary ACh testing and measurement of lactate production. Women were more frequently observed in their study than our study [50% (59/117) vs. 34% (254/746), *P* < 0.001]. Ohba *et al.*^21^ reported that ES was diagnosed in 216 patients (58%), and microvascular coronary artery spasm was recognized in 50 patients (14%) among 370 patients who had unobstructed coronary artery disease and had an intracoronary ACh-provocation test and measurements of lactate during coronary circulation and quantitative coronary blood flow using a guidewire. The incidence of CMS in their cohort was two times higher than in ours. Furthermore, women comprised 90% of their cases of CMS. However, more women were included in their study than in our study [57% (211/370) vs. 34% (254/746), *P* < 0.001]. Suda *et al.*^22^ reported that ES was observed in 128 patients (68%) and 22 patients (12%) were diagnosed with CMS among 187 patients with angina-like chest pain and unobstructed coronary arteries by using intracoronary ACh vasoreactivity testing and guidewires. Women comprised 74 patients (40%) in their study. The female incidence in the study by Suda *et al.* was slightly higher than ours but not significantly so [40% (74/187) vs. 34% (254/746), *P* = 0.1571]. In contrast, Ong *et al.*^23^ reported the incidence of ES and CMS using intracoronary ACh injection test among 124 Caucasian patients with stable angina pectoris and unobstructed coronary arteries. The ACh test revealed ES in 35 patients, while CMS was diagnosed in 42 patients by intracoronary injection of ACh. Aziz *et al.*^24^ also reported the frequency of ES and CMS using an intracoronary ACh injection test among 1379 consecutive Caucasians with unobstructed coronary artery disease. Although the data were duplicated among 13 months in all study periods (88 months), the ACh test revealed ES in 355 patients (26%), and CMS was observed in another 458 patients (33%). Schoenenberger *et al.*^25^ reported that a total of 33% (286 patients) had CMS, 20% (198 patients) had ES, and 7% (53 patients) had a combination of both by using a single ACh dose. According to report by Ford *et al.*,^26^ among 81 patients with unobstructed coronary artery disease, 59 patients were diagnosed with CMS and 11 patients were diagnosed with ES. Approximately 70% of their study subjects were women. The CorMicA trial reported that ES was diagnosed in 56 patients (37%) and CMS was observed in 109 patients (72%) among 151 patients with unobstructed coronary artery disease by using guidewire and ACh bolus testing.^27^ Moreover, 31 patients (21%) had both ES and CMS. Compared with Western studies, the frequency of women and CMS in Japanese populations by ACh testing is scarce, while the frequency of ES in Japanese populations is markedly higher than that in Western studies, as shown in Supplementary material online, *Table S4*. The median ratio of ES to CMS in Western study subjects with unobstructed coronary arteries was significantly lower than that in Japanese populations [0.68 (interquartile range: 0.19 to 0.83) vs. 5.22 (interquartile range: 2.17 to 8.23), *P* < 0.05]. The ratio of ES to CMS was <1.0 in Western reports, whereas this ratio in Japanese studies was over 2.0. This difference may be a racial difference or a procedural disparity. Female patients were common among Japanese patients with CMS as well as in Western populations, while ES was markedly more common among males except Western studies. However, there were some methodological differences, including maximal ACh doses and administration time, between the Western and Japanese studies.

### Intracoronary acetylcholine testing (20 s of injection vs. 3 min of injection)

Japanese Circulation Society guidelines recommend intracoronary ACh testing for over 20 s of injection with a temporary pacemaker. In 1986, the usefulness of intracoronary ACh testing in patients with variant angina was reported, and the administration of intracoronary incremental ACh 20–100 µg reproduced ST-elevation in more than 90% of cases of spontaneous ST-elevation.^7^ This ACh testing method was developed for the documentation of coronary artery spasms.^28^^,^^29^ In contrast, some Caucasian cardiologists employed over 3 min of ACh administration (2, 20, 100, and 200 µg) into the LCA without a pacemaker. This ACh method was derived from the coronary endothelial dysfunction study (Evaluation of Nifedipine and Cerivastatin On Recovery of coronary Endothelial function: ENCORE I).^30^ This ACh testing method was developed for the investigation of a coronary endothelial dysfunction but not coronary spasms. Furthermore, this ACh 3-min administration is still not verified for the reproduction of coronary spasms in patients with vasospastic angina or variant angina. Coronary artery spasms may be involved in the pathogenesis of coronary endothelial dysfunction and coronary smooth muscle dysfunction. We performed both ACh tests in the same 30 patients into the LCA.^31^ However, the incidence of ES, chest symptoms, and ischaemic ECG changes during ACh 20-s injection was significantly higher than that during ACh 3-min administration. These differences might also be influenced by the short expression time of eNOS as well as the short half-life of ACh. Moreover, we could not identify CMS in 30 Japanese patients, although ES was found in 10 patients during ACh 3-min injection. Intracoronary administration time plays a key role in provoked epicardial and microvascular spasms.

### Clinical restriction of acetylcholine spasm provocation tests

We defined positive ES as ≥90% narrowing and usual chest symptoms and ischaemic ECG changes. In the 113 patients with the unclassified ACh test and ≥90% narrowing, 63 patients complained of usual chest symptoms without ischaemic ECG changes. Only ≥90% stenosis without chest symptoms or ischaemic ECG changes was observed in 18 patients, while 13 patients had ≥90% stenosis and ischaemic ECG changes without chest symptoms. Furthermore, 16 patients complained of unusual chest symptoms and had ≥90% stenosis and ischaemic ECG changes, while 3 patients with ≥90% stenosis complained of unusual chest symptoms without ECG changes. Because the effect of intracoronary bolus injection of ACh for 20 s is short, chest symptoms and ischaemic ECG changes were not always documented when we observed ≥90% stenosis in each coronary artery. In contrast, continuous intracoronary injection of ACh for 3 min may affect the occurrence of abnormal endothelial dysfunction. Individual sensations of chest pain after intracoronary ACh injection are a clinical limitation. According to our previous report, ∼7% of patients who had ≥90% stenosis after intracoronary ACh injection for 20 s based on the JCS guidelines had neither chest symptoms nor ischaemic ECG changes.^32^ According to the ENCORE I study at baseline, transient ECG changes and the occurrence of chest pain were observed in 11 (3%) and 3 patients (1%), respectively, although complete coronary occlusion occurred with all three doses of ACh in 42 patients (13%). Furthermore, in the ENCORE II study, just 5 patients (1%) had transient ECG changes among the 427 baseline study subjects although 116 patients (28%) could not receive all three doses of ACh possibly due to coronary constriction or occlusion.^33^ According to the report by Ong *et al.*, the ACh test was inconclusive in 242 patients (29%). Reproduction of symptoms was found in 87 patients (36%), and ischaemic ECG changes alone were observed in 81 patients (33.5%) with inconclusive ACh tests, whereas ES (≥75% diameter reduction) was revealed in 74 patients (31%), including 40 with associated ischaemic ECG changes but no reproduction of symptoms, one with reproduction of symptoms but without ECG shifts, and 33 with neither chest symptoms nor ischaemic ECG changes. We also diagnosed 26% of the study subjects with an unclassified ACh test in this study.

### Comparisons of diagnosing coronary microvascular dysfunction using a guidewire and acetylcholine testing

According to the Western guidelines, coronary microvascular dysfunction should be diagnosed by using a guidewire first.^34^^,^^35^ Cardiologists tend to neglect the presence of ES and CMS when they perform intracoronary ACh testing. Furthermore, Western studies do not always perform spasm provocation testing on either coronary artery.^34^^,^^36^^,^^37^ In this study, we identified 10 patients with CMS in only RCAs among all 40 patients with CMS. Considering the economic cost, it may be better for all cardiologists to perform intracoronary ACh testing before using a guidewire when they test patients for ES and CMS. Intracoronary ACh testing is safe and a clinically useful method to diagnose patients with ES and CMS.

### Study limitations

We had several limitations of this study. One is that this is a retrospective, single-centre, small study. Second, we could not perform perfect ACh tests on either coronary artery. However, we could perform ACh tests on both coronary arteries in 96% of all study subjects. Third, female patients accounted for just 34% of the study subjects, although we recruited consecutive patients. According to previous Caucasian studies, women made up more than half of the patients. Patient’s selection bias may be concern. Fourth, the incidence of complications during ACh testing was higher than that in Caucasian populations. However, there were no irreversible complications in this study. Fifth, we could not analyse the ischaemic ECG changes during ACh testing in some patients due to pacemaker rhythm. Sixth, the ratio of ES to CMS in our study was higher than that in other Japanese studies, possibly due to the use of consecutive populations and fewer female patients. Further study is necessary to clarify the racial differences in ES and CMS between Caucasian and Asian populations, including Japanese populations.

## Conclusions

The incidence of ES was 44%, while the frequency of CMS was only 5%, in consecutive Japanese patients who had unobstructed coronary arteries using intracoronary ACh testing based on the JCS guidelines. Furthermore, only four patients had CMS coexisting with ES. Coronary microvascular spasm was frequently observed in the LCA than that in the RCA. Compared with Western populations, Japanese patients had higher rates of ES and fewer cases of CMS.

## Lead author biography

**Figure:**
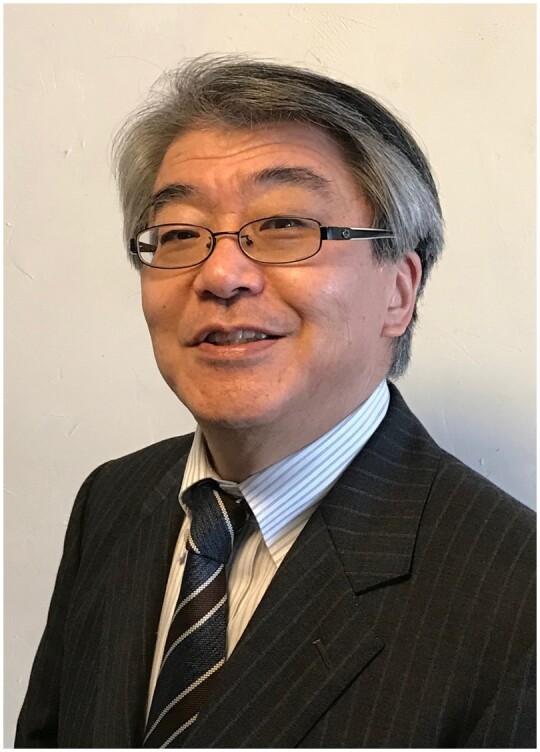


I have been interested in coronary artery spasm for more than 30 years. I have performed spasm provocation tests more than 3000 cases including 1800 acetylcholine testing and 1200 ergonovine tests. I have learned many things from these vasoreactivity tests. Japanese Circulation Society guideline and COVADIS group defined spasm provocation test as class I, while ACC/AHA guideline and ESC guideline classified spasm provocation test as class IIb and class IIa, respectively. Vasoreactivity testing is essential for diagnosing patient with epicardial spasm and coronary microvascular spasm. Spasm provocation test should be classified as class I in all over the world.

## Supplementary material

Supplementary material is available at *European Heart Journal Open* online.

**Conflict of interest:** none declared. 

## Supplementary Material

oeab012_Supplementary_Data

## Notes

I have been interested in coronary artery spasm for more than 30 years. I have performed spasm provocation tests more than 3000 cases including 1800 acetylcholine testing and 1200 ergonovine tests. I have learned many things from these vasoreactivity tests. Japanese Circulation Society guideline and COVADIS group defined spasm provocation test as class I, while ACC/AHA guideline and ESC guideline classified spasm provocation test as class IIb and class IIa, respectively. Vasoreactivity testing is essential for diagnosing patient with epicardial spasm and coronary microvascular spasm. Spasm provocation test should be classified as class I in all over the world.
