# Supplementary material for: Intracoronary acetylcholine testing among 746 consecutive Japanese patients with angina-like chest pain and unobstructed coronary artery disease
Source: Eur Heart J Open. 2021 Aug 11;2(1):oeab012. doi: 10.1093/ehjopen/oeab012 (PMC9242056; doi:10.1093/ehjopen/oeab012)
Supplement: oeab012_Supplementary_Data [file oeab012_supplementary_data.doc]

**Supplementary file Table S1**

**Acetylcholine testing results in 40 patients with coronary microvascular spasm**

|  |  |  |  |  |  |  |  |  |  |  |  |
| --- | --- | --- | --- | --- | --- | --- | --- | --- | --- | --- | --- |
|  |  |  |  | **RCA** |  |  |  | **LCA** |  |  |  |
| **No** | **Age** | **Sex** | **Chest symptom** | **Dose (μg)** | **Chest symptom** | **ST shift** | **Spasm** | **Dose (μg)** | **Chest symptom** | **ST shift** | **Spasm** |
|  |  |  |  |  |  |  |  |  |  |  |  |
|  |  |  |  |  |  |  |  |  |  |  |  |
| 1 | 56 | F | Rest | 50 | no | no | no | 20/50/100 | usual | V4-6 (H, 1.0) | CMS |
| 2 | 75 | F | Rest | 20/50/80 | no | no | no | 50/100 | usual | V2-4 (DS, 2.0) | CMS |
| 3 | 70 | F | Rest | 50 | usual | ST ele in INF (3.0) | CMS | 50/100 | no | no | no |
| 4 | 72 | F | Rest | 80 | usual | Pacing rhythm | 4(d) unclassified | 20/50/100/200 | usual | V4-6 I aVL (DS, 2.0) | CMS |
| 5 | 74 | M | Rest | 50/80 | usual | INF (H, 2.0) | CMS | 100 | no | no | no |
| 6 | 58 | M | Rest | 50/80 | no | no | no | 20/50/100/200 | usual | V4-6 (J, 2.0) | CMS |
| 7 | 65 | M | Rest | 20/50/80 | usual | INF (DS, 1.0) | CMS | 20/50/100/200 | usual | no | 8(d)/11(d) unclassified |
| 8 | 84 | F | Effort | 50 | no | no | no | 20/50/100 | usual | V3-5 (H, 2.0) | CMS |
| 9 | 76 | F | Effort |  |  |  |  | 50/100 | usual | V3-6 (J, 2.0) | CMS |
| 10 | 69 | F | Effort | 50/80 | no | V1-6 (DS, 1.0) | unclassified | 20/50/100/200 | usual | V3-6 (DS, 2.0) | CMS |
| 11 | 85 | F | Effort | 80 | usual | no | unclassified | 100/200 | usual | V3-6 (H, 1.0) | CMS |
| 12 | 66 | F | Effort | 50/80 | usual | INF (H, 1.5) | CMS | 20/50/100/200 | usual | no | unclassified |
| 13 | 83 | F | Effort | hypo |  |  |  | 20/50/100/200 | usual | V4-6 (H, 1.5) | CMS |
| 14 | 70 | M | Effort | 80 | usual | Pacing rhythm | unclassified | 20/50/100/200 | usual | V56 (DS, 1.0) | CMS |
| 15 | 78 | M | Effort | 20/50 | no | no | no | 20/50/100/200 | usual | V4-6 (J, 2.0) | CMS |
| 16 | 58 | M | Effort | 25/50 | no | no | no | 50/100 | usual | V3-6 (DS, 4.0) | CMS |
| 17 | 49 | M | Effort | 50/80 | usual | no | 4(d) unclassified | 50/100/200 | usual | ST ele in INF V56 (2.0) | CMS |
| 18 | 45 | F | R/E | hypo |  |  |  | 20/50/100/200 | usual | V2-6 INF (DS, 1.0) | CMS |
| 19 | 73 | F | R/E | 80 | unusual | no | unclassified | 50/100/200 | usual | V3-6 I aVL (DS,2.0) | CMS |
| 20 | 85 | M | R/E | 25/50/75 | no | no | no | 100 | usual | V2-6 (H, 1.0) | CMS |
| 21 | 83 | M | R/E | 50/80 | usual | V4-6 (H, 1.0) | CMS | 50/100/200 | usual | V4-6 INF (H, 2.0) | CMS |
| 22 | 61 | M | R/E | 4Fr wedge |  |  |  | 20/50/100 | usual | V1-4 (H, 2.0) | CMS |
| 23 | 72 | F | Another | 50/80 | usual | INF (H, 1.0) | CMS | 50/100 | usual | V56 (H, 1.0) | CMS |
| 24 | 50 | F | Another | 20/50/80 | usual | no | unclassified | 20/50/100 | usual | V1-5 (J, 3.0) | CMS |
| 25 | 54 | F | Another | 20/50/80 | usual | IINF (H, 1.0) | CMS | 50/100 | usual | V4-6 INF (H, 1.5) | CMS |
| 26 | 49 | F | Another | 50/80 | usual | INF (H, 1.0) | CMS | 50/100 | no | V56 (H, 1.0) | unclassified |
| 27 | 54 | F | Another | 20/50/80 | usual | INF (J, 2.0) | CMS | 20/50/100 | usual | no | unclassified |
| 28 | 80 | F | Another | 50/75 | no | no | no | 50/100 | usual | V3-5 (H, 1.5) | CMS |
| 29 | 67 | F | Another | 20/50/80 | no | no | no | 20/50/100 | usual | V4-6 (H, 1.0) | CMS |
| 30 | 50 | F | Another | 50/80 | usual | INF (H, 1.0) | CMS | 50/100 | usual | no | unclassified |
| 31 | 77 | F | Another | 20/50 | usual | no | unclassified | 20/50/100 | usual | V4-6 (J, 2.0) | CMS |
| 32 | 80 | F | Another | 25/50 | usual | INF (DS, 3.0) | CMS | 50/100 | no | V4-6 (DS, 3.0) | unclassified |
| 33 | 68 | F | Another | 25/50 | no | no | no | 50/100 | usual | V4-6 (DS, 1.0) | CMS |
| 34 | 69 | F | Another | 20/50 | usual | INF (H, 1.5) | CMS | 50/100 | usual | V2-5 (H, 1.0) | CMS |
| 35 | 58 | F | Another | 20/50 | usual | no | 4(d) unclassified | 50/100 | usual | V4-6 (DS, 2.0) | CMS |
| 36 | 65 | M | Another | 20/50/80 | usual | no | unclassified | 20/50/100/200 | usual | V56 (H, 1.0) | CMS |
| 37 | 70 | F | Another | 20/50/80 | usual | V3-6 (H, 1.0) | 3(f) | 20/50/100/200 | usual | V2-4 (H, 4.0) | CMS |
| 38 | 67 | M | Rest | 20/50/80 | usual | INF (H, 2.0) | CMS | 20 | usual | V2-6 (H, 1.0) | 6(f) |
| 39 | 53 | M | Rest | 20/50 | usual | ST ele in INF (3.0) | CMS | 50/100 | usual | ST ele in V1-4 (3.0) | 6(f) |
| 40 | 62 | M | Rest | 20/50 | usual | ST ele in INF (2.0) | 2(f) | 50/100 | usual | ST ele in V2-4 (3.0) | CMS |
|  |  |  |  |  |  |  |  |  |  |  |  |

(RCA: right coronary artery, LCA: left coronary artery, M: male, F: female, R/E: rest & effort, INF: inferior (II III aVF leads), H: horizontal, J: junctional, DS: down slopping, ele: elevation, CMS: coronary microvascular spasm, f: focal spasm, d: diffuse spasm)

**Supplementary file Table S2**

**Univariable and multivariable analysis between positive spasm and negative result**

|  |  | |  |  | |  | | |  |  | |  |
| --- | --- | --- | --- | --- | --- | --- | --- | --- | --- | --- | --- | --- |
|  |  | **Univariable analysis** | | |  | |  | **Multivariable analysis** | | |  | |
|  |  | |  |  | |  | | |  |  | |  |
|  |  | |  |  | |  | | |  |  | |  |
|  | OR | | 95% CI | p value | | OR | | | 95% CI | p value | |  |
|  |  | |  |  | |  | | |  |  | |  |
|  |  | |  |  | |  | | |  |  | |  |
| Age |  | |  | 0.7021 | |  | | |  |  | |  |
| Sex | 0.525 | | 0.380-1.723 | 0.0000414 | |  | | |  |  | |  |
| History of smoking | 2.198 | | 1.597-3.034 | 0.000000551 | | 1.900 | | | 1.100-3.300 | 0.0192 | |  |
| Hypertension | 0.949 | | 0.698-1.289 | 0.763 | |  | | |  |  | |  |
| Dyslipidemia | 1.221 | | 0.902-1.653 | 0.181 | |  | | |  |  | |  |
| Diabetes mellitus | 0.889 | | 0.609-1.295 | 0.582 | |  | | |  |  | |  |
| LVEF by UCG |  | |  | 0.2931 | |  | | |  |  | |  |
| Calcium channel blocker | 2.687 | | 1.979-3.660 | 0.0000000000358 | | 1.920 | | | 1.360-2.710 | 0.0008 | |  |
| ACEI or ARB | 1.163 | | 0.762-1.778 | 0.475 | |  | | |  |  | |  |
| Nitrate or nicorandil | 2.706 | | 1.975-3.724 | 0.0000000000844 | | 1.770 | | | 1.240-2.520 | 0.0016 | |  |
| Beta-blocker | 0.673 | | 0.360-1.237 | 0.197 | |  | | |  |  | |  |
| Statin | 1.467 | | 0.995-2.174 | 0.0476 | |  | | |  |  | |  |
|  |  | |  |  | |  | | |  |  | |  |

(LVEF: left ventricular ejection fraction, UCG: ultrasonography, ACEI: angiotensin converting enzyme inhibitor, ARB: angiotensin receptor blocker)

**Supplementary file Table S3**

**Univariable and multivariable analysis between epicardial spasm and microvascular spasm**

|  |  | |  |  | |  | | |  |  | |  |
| --- | --- | --- | --- | --- | --- | --- | --- | --- | --- | --- | --- | --- |
|  |  | **Univariable analysis** | | |  | |  | **Multivariable analysis** | | |  | |
|  |  | |  |  | |  | | |  |  | |  |
|  |  | |  |  | |  | | |  |  | |  |
|  | OR | | 95% CI | p value | | OR | | | 95% CI | p value | |  |
|  |  | |  |  | |  | | |  |  | |  |
|  |  | |  |  | |  | | |  |  | |  |
| Age |  | |  | 0.1441 | |  | | |  |  | |  |
| Sex | 6.470 | | 3.075-14.15 | 0.0000000822 | |  | | |  |  | |  |
| History of smoking | 0.175 | | 0.081-0.366 | 0.000000533 | | 0.188 | | | 0.093-0.379 | 0.001 | |  |
| Hypertension | 1.435 | | 0.699-2.932 | 0.306 | |  | | |  |  | |  |
| Dyslipidemia | 0.488 | | 0.222-1.021 | 0.0446 | |  | | |  |  | |  |
| Diabetes mellitus | 1.448 | | 0.597-3.252 | 0.395 | |  | | |  |  | |  |
| LVEF by UCG |  | |  | 0.9643 | |  | | |  |  | |  |
| Calcium channel blocker | 0.503 | | 0.245-1.031 | 0.0527 | |  | | |  |  | |  |
| ACEI or ARB | 2.554 | | 1.103-5.631 | 0.0199 | |  | | |  |  | |  |
| Nitrate or nicorandil | 0.338 | | 0.147-0.726 | 0.0025 | | 0.396 | | | 0.187-0.842 | 0.016 | |  |
| Beta-blocker | 2.783 | | 0.751-8.599 | 0.0638 | |  | | |  |  | |  |
| Statin | 0.905 | | 0.344-2.120 | 1 | |  | | |  |  | |  |
|  |  | |  |  | |  | | |  |  | |  |

(LVEF: left ventricular ejection fraction, UCG: ultrasonography, ACEI: angiotensin converting enzyme inhibitor, ARB: angiotensin receptor blocker)

**Supplementary file Table S4**

**Comparisons of vasoreactivity testing between Western and Japanese study in patients with nonobstructive coronary artery disease**

|  |  |  |  |  |  |  |  |
| --- | --- | --- | --- | --- | --- | --- | --- |
|  | **Diagnosis** | **Vasoreactivity test**  **(ACh dose (μg))** | **Number** | **Female** | **ES** | **CMS** | **ES/CMS** |
|  |  |  |  |  |  |  |  |
|  |  |  |  |  |  |  |  |
| Ong et al.^22)^ | NOCAD | LCA (2/20/100/200) RCA (80) | 124 | 87 (70) | 35 (28) | 42 (34) | 0.83 |
| Aziz et al.^23)^ | NOCAD | LCA (2/20/100/200) RCA (80) | 1379 | 806 (58) | 355 (26) | 458 (33) | 0.78 |
| Schoenenberger et al.^24)^ | NOCAD | RCA/LCA (64) | 718 | 361 (50) | 198 (28) | 286 (40) | 0.69 |
| Ford et al.^25)^ | NOCAD | LCA (100) RCA (50) | 81 | 56 (69) | 11 (14) | 59 (73) | 0.19 |
| The CorMicA Trial^26)^ | NOCAD | LCA (100) RCA (50) | 151 | 111 (74) | 25 (17) | 78 (52) | 0.32 |
| **Total (Western)** |  |  | **2453** | **1421 (58)** | **624 (25)** | **923 (38)** | **0.68** |
|  |  |  |  |  |  |  |  |
| Mohri et al.^19)^ | NOCAD | LCA (10/30100)  RCA (5/15/50) | 117 | 59 (50) | 63 (54) | 29 (25) | 2.17 |
| Ohba et al.^20)^ | NOCAD | LCA (20/50/100)  RCA (50) | 370 | 211 (57) | 216 (58) | 50 (14) | 4.32 |
| Suda et al.^21)^ | NOCAD | LCA (20/50/100)  RCA (20/50) | 187 | 74 (40) | 128 (68) | 22 (12) | 5.82 |
| Sueda et al. | NOCAD | LCA (20/50/100/200) RCA (20/50/80) | 746 | 254 (34) | 329 (44) | 40 (5) | 8.23 |
| **Total (Japanese)** |  |  | **1420** | **598 (42)**** | **736 (52)**** | **141 (10)**** | **5.22*** |
|  |  |  |  |  |  |  |  |

(NOCAD: nonobstructive coronary artery disease, MINOCA: myocardial infarction with nonobstructive coronary artery, ES: epicardial spasm, CMS: coronary microvascular spasm, ACh: acetylcholine, ER: ergonovine, LCA: left coronary artery, RCA: right coronary artery, *: P<0.05 & **: p<0.001 vs. European total stud
